# Under-Treatment of Older Patients with Newly Diagnosed Epithelial Ovarian Cancer Remains an Issue

**DOI:** 10.3390/cancers13050952

**Published:** 2021-02-25

**Authors:** Lucy Dumas, Rebecca Bowen, John Butler, Susana Banerjee

**Affiliations:** 1 Gynaecology Unit, Royal Marsden NHS Foundation Trust, London SW3 6JJ, UK; lucy.dumas@nhs.net (L.D.); john.butler@cancer.org.uk (J.B.); 2 Institute of Cancer Research, 15 Cotswold Road, Sutton, London SM2 5NG, UK; 3 Department of Oncology, Royal United Hospitals Bath NHS Foundation Trust, Bath BA1 3NG, UK; rebecca.bowen3@nhs.net

**Keywords:** ovarian cancer, older, treatment

## Abstract

**Simple Summary:**

Cancer treatment and survival in older women is topical and important in an era where the proportion of older adults being treated for cancer in the clinic is rising. As survival outcomes in older women continue to lag behind those of younger and middle-aged women, further investigation is required into current treatment practices to identify target areas to address this deficit. We present treatment patterns, tolerance and outcomes of 280 women aged 65 and above treated for newly diagnosed ovarian cancer at two UK cancer centres between 2009 and 2015. We demonstrate that older women continue to receive lower rates of standard care first line therapy. When adjusted for stage at diagnosis, surgical outcome and chemotherapy given, age was not an independent risk factor for poorer overall survival.

**Abstract:**

Older women with ovarian cancer have disproportionately poorer survival outcomes than their younger counterparts and receive less treatment. In order to understand where the gaps lie in the treatment of older patients, studies incorporating more detailed assessment of baseline characteristics and treatment delivery beyond the scope of most cancer registries are required. We aimed to assess the proportion of women over the age of 65 who are offered and receive standard of care for first-line ovarian cancer at two UK NHS Cancer Centres over a 5-year period (December 2009 to August 2015). Standard of care treatment was defined as a combination of cytoreductive surgery and if indicated platinum-based chemotherapy (combination or single-agent). Sixty-five percent of patients aged 65 and above received standard of care treatment. Increasing age was associated with lower rates of receiving standard of care (35% > 80 years old versus 78% of 65–69-year-olds, *p* = 0.000). Older women were less likely to complete the planned chemotherapy course (*p* = 0.034). The oldest women continue to receive lower rates of standard care compared to younger women. Once adjusted for Federation of Gynaecology and Obstetrics (FIGO) stage, Eastern Cooperative Oncology Group (ECOG) performance status and first-line treatment received, age was no longer an independent risk factor for poorer overall survival. Optimisation of vulnerable patients utilising a comprehensive geriatric assessment and directed interventions to facilitate the delivery of standard of care treatment could help narrow the survival discrepancy between the oldest patients and their younger counterparts.

## 1. Introduction

Ovarian cancer (including primary peritoneal and fallopian tube cancer) is predominantly diagnosed in older women with around half of all new diagnoses occurring in women over the age of 65. Older patients are less likely to be enrolled in clinical trials [1,2] that go onto to shape current gold standards. Treatment decisions are usually based on clinical trial results, which include a younger, less frail population and are applied to an older and often less well group. The efficacy and tolerability of standard of care and novel therapies in an older, potentially frailer population are therefore not clearly understood.

It has long been shown that survival outcomes are disproportionately lower in older patients [3] and delayed diagnosis/late presentation [4], more advanced disease at diagnosis [5,6,7], higher rates of emergency presentation [8], higher rates of unclassified or unclassifiable tumours [3] as well as lower physical performance status and higher prevalence of medical and functional comorbidities [9] contribute to this. Recent studies have reported high rates of no recorded treatment in older patients, for example 60% of ovarian cancer patients aged over 79 had no record of any treatment in England between 2016 and 2018 [10,11]. Developing our understanding of the “real-world” experience of treatment for ovarian cancer in an older population is necessary. Until the reasons for the difference in survival between older and younger women are more clearly understood, efforts to address the gaps and improve outcomes in our older population will be hampered. Large-scale cancer registry data by both the EUROCARE [12,13,14,15] series and the International Cancer Benchmarking Partnership (ICBP) [6,7,16,17] demonstrate that both short and long-term survival outcomes in older women continue to be significantly inferior to those seen in middle-aged and younger women [12,17,18]. Notably, an improving trend in 1- and 5-year survival for all age groups has been reported excepting those aged over 75 years [19].

The field of geriatric oncology has rapidly developed over the last two decades. The core principles outline the need to holistically assess patients using a comprehensive geriatric assessment rather than basing treatment decisions purely on chronological age. Both the International Society of Geriatric Oncology (SIOG) [20] and the American Society of Clinical Oncology (ASCO) [21] have now recommended that geriatric assessment (GA) be undertaken in all adults aged 65 years and over being considered for systemic anti-cancer therapy. Crucially, clinicians should implement GA-directed interventions in order to optimise patient care. 

## 2. Results

### 2.1. Patient Baseline Characteristics

Two hundred and eighty patients met the inclusion criteria. Patients were divided into four age cohorts (65–69 years, 70–74 years, 75–79 years and >80 years). The majority (76%) of patients had stage 3 or 4 disease at presentation (Table 1). Stage distribution did not alter with increasing age (*p* = 0.293). 29% of patients were ECOG performance status 2 or 3. Increasing age was significantly associated with a worsening ECOG performance status (*p* = 0.008). Forty-nine percent of patients over the age of 80 were PS 0 or 1 compared to 70.4% of patients in the 65–69 years cohort (Table 1). The majority (69.6%) of patients were diagnosed with high-grade serous carcinoma. Histological subtype did not vary according to age (*p* = 0.547). 

The most commonly documented comorbidities were cardiovascular disease (27.5%), hypertension (40.4%), respiratory disease (10%) and diabetes (10.4%). Polypharmacy at the initial consultation, defined as taking 3 or more daily prescribed medications, was present in 40% of patients. Neither cardiovascular disease nor hypertension was associated with increasing age. 48.9% women were anaemic (any grade) at baseline with 11.4% patients having a Grade 2 or higher anaemia. Impaired renal function at the start of treatment was also common with 37% of all patients having at least a mild-moderate reduction of glomerular-filtration rate (GFR) of 60 mL/min or less, amounting to chronic kidney disease grade 3. A total of 40.7% patients had an albumin below 35 g/L at baseline and 22.5% of patients had an albumin less than 30 g/L. Hypoalbuminaemia was not associated with increasing age (*p* = 0.36) (Table 2). Factors and comorbidities significantly associated with advancing age were polypharmacy (*p* = 0.01), respiratory disease (*p* = 0.007) and cognitive impairment (*p* = 0.001) (Table 2.) Increasing age was associated with a higher proportion of women living alone (51% of those >80 years compared with 22% of those aged 65–69 years, *p* = 0.000). Older women were also significantly more likely to live in supported accommodation (*p* = 0.032), use a walking aid (*p* = 0.026) or have a degree of visual impairment (*p* = 0.016). A quarter of all patients reported reduced activities of daily living in the weeks and months preceding their diagnosis. Self-reported weight loss was also prevalent with 23.6% of patients reporting weight loss over the 3 months prior to their diagnosis (Table 2.)

### 2.2. First Line Treatment

**Figure 1:**
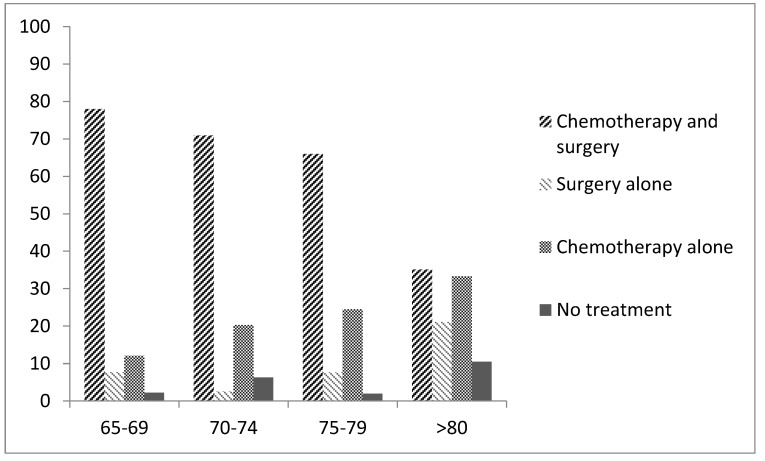
**First**-line treatment received according to age cohort.

Sixty-five percent of patients received standard of care cytoreductive surgery and platinum-based chemotherapy in keeping with the European Society of Medical Oncolgogy ESMO)-European-Society of Gynaecological Oncology (ESGO) consensus recommendations [22]. Increasing age was associated with reducing rates of receiving standard of care therapy with 35.1% of those over the age of 80 receiving both chemotherapy and surgery compared to 78% in those aged 65–69 years (*p* = 0.000). Ten percent of patients over the age of 80 received no cancer treatment (Figure 1). Six (2%) patients declined surgery and three (1%) declined chemotherapy. Increasing age was associated with lower rates of undergoing cytoreductive surgery (*p* = 0.001) as well as complete cytoreduction (defined according to the post-operative report) (*p* = 0.006) with complete cytoreduction obtained in only 28% of those over the age of 80 compared to 69% in those aged 65–69. When optimal cytoreduction (<1 cm residual disease) is reported as a proportion of those patients who underwent surgery, the rates of optimal cytoreduction was 76% in all age cohorts apart from those over 80 where it was 49%. 53.7% of women received standard carboplatin and paclitaxel chemotherapy as first-line treatment. Older women were less likely to receive doublet chemotherapy (19.3% in those over the age of 80 compared to 73.6% in those aged 65–69 years, *p* = 0.000). Overall, 7.8% women aged 65 and above any form of targeted therapy during first-line treatment. This proportion decreased with advancing age (2.3% of women over the age of 80 compared to 12.4% women between the ages of 65–69 received some form of targeted therapy during first-line treatment (*p* = 0.05). 

Subsequently, the primary treatment of only those women with advanced (Federation of Gynaecology and Obstetrics (FIGO) stage III/IV) disease was assessed. A total of 62.9% of women aged 65 and over received standard of care, with this proportion decreasing significantly with increasing age (*p* = 0.000). 46.3% of women aged 80 years and over underwent cytoreductive surgery. In these women complete cytoreduction was achieved in 31.6%, compared to those aged between 65–69 years of whom 82.4% underwent surgery, and 69.6% had complete cytoreduction (*p* = 0.014). 

### 2.3. Treatment Tolerance

Overall, 27.3% of patients developed a grade two or higher haematological toxicity. Neutropenia was more common in younger patients (69.6% in those aged 65–69 vs. 18.8% in those aged 75–79, *p* = 0.007). Older patients did not experience higher rates of severe haematological toxicity (*p* = 0.554). However, increasing age was associated with a trend towards a higher rate of G3 or 4 non-haematological toxicities although this did not reach statistical significance (32.5% vs. 13.4% in those aged >80 years vs. those aged 65–69 years, *p* = 0.082). Increasing age was significantly associated with a lower likelihood of completing 6 cycles (*p* = 0.034). Of the 38 (15.8%) women who discontinued treatment early, 21 (55%) did so because of toxicity. Discontinuation due to toxicity was higher in older patients, for example 54.5% of 75–79-year-olds compared to 36.4% of those aged 65–69 years, although this did not reach statistical significance (*p* = 0.15). 28.5% of all patients were admitted to hospital as an emergency at some stage during their primary treatment with no variation due to age (*p* = 0.135), 30-day mortality was 1.24% across the whole cohort and did not vary according to age (*p* = 0.184) (Table 3).

### 2.4. Treatment at Relapse

At first relapse, 50.4% of women received chemotherapy; however, older women were significantly less likely to receive second-line chemotherapy at progression. A total of 35.5% of women over the age of 75 received chemotherapy at relapse, compared to 62.5% of those aged 65–69 years (*p* = 0.021). One patient (aged 75 years) underwent secondary debulking surgery. Seventy-five women (59% of those who had treatment for relapsed disease) received carboplatin-based chemotherapy at first relapse. Of those who received chemotherapy at first relapse, 56 (45%) received a carboplatin doublet regimen (paclitaxel, pegylated liposomal doxorubicin or gemcitabine with or without a targeted agent for example, bevacizumab). Nineteen (15%) women received single-agent carboplatin. Of those who received platinum at first relapse, 65% achieved some degree of tumour shrinkage as their best response according to the local radiological report with 89% achieving at least stable disease. In those patients who received non-platinum containing regimens, 21 patients (15%) received weekly paclitaxel resulting in a 33.3% radiological response rate and a 52% clinical benefit rate (defined as patients who achieved at least stable disease as their best response documented). Twenty-three (18.9%) patients received either pegylated liposomal doxorubicin or doxorubicin. No responses were seen in this group although 6 (26%) patients had stabilisation of their disease.

### 2.5. Survival Outcomes

**Figure 2:**
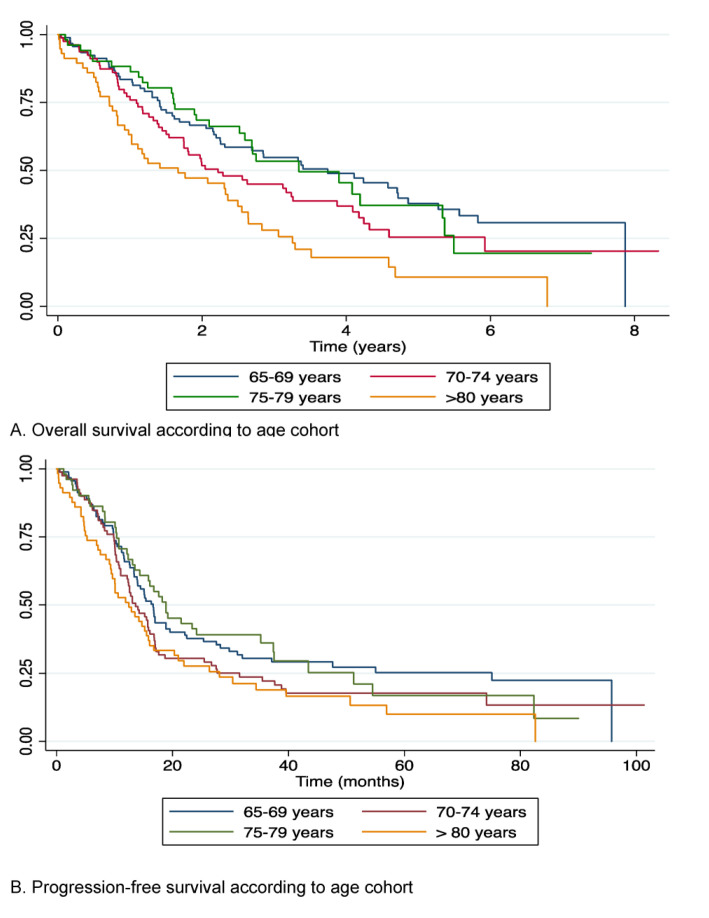
(**A**) Overall survival according to age cohort. (**B**) Progression-free survival according to age cohort.

Median overall survival (OS) for all patients was 31.5 months. For patients diagnosed with stage III and stage IV disease, median OS was 28.3 and 14 months respectively. 1-year and 5-year survival was 78.1% (95% CI 72.7–82.5) and 28.7% (95% CI 22.5–35.2) respectively. Overall survival was broadly equivalent over the first three age cohorts however patients over the age of 80 had a significantly lower survival than those aged 65–69 years (median OS 20.02 months vs. 44.91 months, *p* = 0.000) (Figure 2). First line carboplatin/paclitaxel combination chemotherapy was associated with improved survival outcomes compared to single-agent carboplatin (OS 39.5 vs. 30.6 months), those patients who received no chemotherapy had an OS of 9.7 months (*p* = 0.003). Progression-free survival (PFS) was similar across all age groups up to the age of 80 but patients aged 80 years and over had a median PFS of 12.3 months compared to 16.4 in those aged 65–69 years (HR 2.0 *p* = 0.00) (Figure 2). 

In univariate analysis, age over 80 years at diagnosis, FIGO stage III/IV disease, incomplete cytoreduction and an ECOG PS of greater than 1 were all associated with poorer survival outcomes. Of the baseline factors and comorbidities collected, the presence of cardiovascular disease (*p* = 0.043), polypharmacy (*p* = 0.011) or having a current or past history of smoking (*p* = 0.008) were all associated with poorer survival outcomes. Requiring assistance with activities of daily living (ADL) (*p* = 0.000), reporting reduced ADLS (*p* = 0.000) and weight loss at diagnosis (*p* = 0.015) were associated with poorer survival outcomes (Table 4). Of the biochemical parameters collected, having any degree of hypoalbuminaemia (*p* = 0.000) or baseline haemoglobin of less than 110 g/L (*p* = 0.000) were associated with poorer survival outcomes. GFR was associated with poorer survival as a continuous variable (*p* = 0.036) however using a threshold of a GFR of 60 mL/min (CKD 3) was not associated with poorer survival outcomes (*p* = 0.064) (Table 4). 

A cox proportional hazards multivariate model was built including treatment-related factors that were predictive, by univariate analysis for overall survival. When adjusted for FIGO stage, surgical outcome, chemotherapy treatment and completion of chemotherapy, age over 80-years-old was no longer an independent risk factor for poorer overall survival. Completion of chemotherapy remained independently associated with overall survival where single-agent versus platinum-doublet chemotherapy was not associated with a significantly different in overall survival in either univariate or multivariate analysis (Table 5). 

## 3. Discussion

This study provides a useful insight into the current real-world treatment of older women diagnosed with epithelial ovarian cancer in two UK cancer centres. There were very low rates of unclassifiable tumours in this series compared to national cancer registry data where over 50% of women over the age of 80 had an unclassified epithelial or miscellaneous tumour [3]. The lack of relationship between unclassifiable tumours and increasing age suggests either an improvement in the approach to the diagnostic process in older patients with more women having a true histological diagnosis being pursued, or the importance of cancer centre management of presumed ovarian cancer. Delayed time to diagnosis and therefore later stage at diagnosis has also been postulated as a cause for poorer survival rates however, stage distribution also did not vary with age in this population with the majority of women of all ages being diagnosed with stage 3 and 4 disease. 

In this series, older patients were more likely to have a poorer ECOG performance status however it is well recognised that ECOG performance status alone is a crude measure in an elderly population that does not accurately reflect the functional and comorbid status of older patients [23,24] and it has also been previously shown that poor performance status should not necessarily preclude first-line treatment in epithelial ovarian cancer due to the high response rates observed to platinum-based chemotherapy [25]. Although many older women maintain fit and active lives, a quarter of the study population reported reduced activities of daily living in the preceding weeks and months before their diagnosis. A significant proportion of women in this study also reported living alone, whilst not a concern in and of itself, living alone without sufficient social network or community support particularly in the context of frailty is a challenge for both patients and oncologists when systemic anti-cancer therapy is being considered.

The most striking difference between the oldest patients and those younger than 80 years was that seen in primary treatment received. Under-treatment has long been postulated as one of the primary reasons for the poorer outcomes in older patients. A large retrospective study in France assessed the impact of age on treatment and survival outcomes whether or not guideline-recommendations for therapy were followed between 1997 and 2011. Women 70 years and over compared to those younger were less likely to undergo surgery (60.9% versus 89.6%, *p* < 0.0001) or receive chemotherapy (57.4% versus 76.4%, *p* < 0.0001). Only 31.9% of patients 70 years and over underwent both surgery and chemotherapy [26]. A prospective study (OVCAD) that included 275 women treated for primary ovarian cancer between 2005 and 2008 also showed that older women were less likely to receive optimal therapy and had poorer progression-free and overall survival. In multivariate analysis, age was an independent risk factor for poorer overall but not progression-free survival [27]. Our findings confirm that older women continue to receive less treatment than their younger and middle-aged counterparts and this is likely to be a significant factor in explaining the poorer outcomes seen in this population. A limitation of this work is that it was not possible from this retrospective study to ascertain whether, in those patients who did not receive either surgery or chemotherapy whether this decision was patient or clinician-led. Documentation of the rationale for a decision for not treatment should be consistently recorded in patient records. In addition, interview studies of the multidisciplinary team making treatment decisions may shed more light on the rationale for patients not receiving standard of care. It has previously been shown that older women desire cure as much as their younger counterparts and are more willing to undergo potentially disfiguring surgery to achieve this than younger patients [28]. In work also undertaken by our group (manuscript under review), we report that older patients desired active treatment and did not consider their age to be a hindrance.

The difference in survival for the oldest patients becoming no longer statistically significant once FIGO stage, surgical outcome and crucially, chemotherapy received are incorporated into the model provides further evidence that if even the oldest patients receive optimal therapy, survival outcomes are comparable. It has been shown that medical and social optimisation of older patients prior to and during systemic anti-cancer therapy can improve chemotherapy completion rates [29]. This approach is being tested in a wider scale in both the PREPARE [30] and GIVE (NCT02785887) studies; these potentially practice-changing results are awaited. We report here that older patients were less likely to receive targeted therapy, however, the only targeted therapy available during the study period was bevacizumab, which only received NICE approval in 2013 (i.e., the final two years of the study period) and thus these rates may not be fully representative. 

Haematological toxicity rates were comparable across the age groups however increasing age was associated with a trend towards a higher rate of non-haematological toxicities. Increasing age was associated with higher early treatment discontinuation rates. This is in keeping with post-hoc analysis from the first-line phase 3 AGO-OVAR3 study, which also showed that women 70 years and over experienced comparable rates of toxicity but were more likely to discontinue treatment early [31]. The AGO-OVAR authors in 2007 suggested a potential difference in attitude towards the treatment of older adults. It can be postulated that this difference persists today. It was relatively rare for patients in our study to decline treatment with six patients declining surgery and three declining chemotherapy; however, the more nuanced decision-making over reducing treatment intensity and early treatment cessation is difficult to reliably elucidate retrospectively. A recent study from the Netherlands reported no treatment rates of 16%; in 40% of these cases it was patient choice, and in 29% it was poor condition in the opinion of the physician [32]. The perspectives of older women on treatment intensity, tolerance and treatment goals are worthy of further study, as the reasons for the reduced treatment intensity remain unclear. 

## 4. Materials and Methods 

Local study approvals were received from the Royal Marsden NHS Foundation Trust and The Royal United Hospitals Bath NHS Foundation Trust (SE486). This was a retrospective observational evaluation of all women over the aged 65 and over treated consecutively for newly diagnosed epithelial ovarian cancer (including tubal and primary peritoneal) over a 5-year period (December 2009 to August 2015) in two UK NHS Cancer Centres. Standard of care treatment was defined as undergoing cytoreductive surgery at any stage in the primary treatment pathway in combination with platinum-based chemotherapy. Details of treatment received, medical comorbidities, polypharmacy, functional level at baseline (where possible) as well as routinely assessed haematological and biochemical parameters were collected. Where toxicities had not been graded in real-time, according to the description of the event, retrospective grading was applied using CTCAE v4.0 for all grade haematological and grade ≥3 non-haematological toxicities.

The primary objective was to assess the proportion of women over the age of 65 who are offered and receive standard of care first-line management. Secondary objectives included assessment of progression-free and overall survival from first diagnosis and first relapse; proportion of patients who suffered a severe haematological or non-haematological chemotherapy toxicity; proportion of patients who received treatment for relapsed disease; rate of hospitalisation and 30-day mortality during chemotherapy. Patients were considered eligible if they were aged 65 years or older at the time of a first new patient appointment with a histologically or cytologically confirmed diagnosis of epithelial ovarian, primary peritoneal and fallopian tube carcinoma at either institution.

### Statistical Considerations 

Chi squared test was used to compare patient baseline characteristics and treatment patterns according to age. Progression-free survival was measured from start of treatment to date of progression or death from any cause. Overall survival was defined as the time from date of diagnosis or date of relapse (depending on the endpoint) to death. Patients without an event were censored at last follow up. Data were censored on the 1 August 2016. Survival outcomes were estimated using the Kaplan–Meier method. Hazard ratios for survival, adjusted for factors likely to be of significance such as age, stage and treatment received were calculated using a cox proportional hazards model. All tests are two sided. A *p*-value of < 0.05 was used to determine statistical significance. All statistical analyses were performed using Stata IC v15.

## 5. Conclusions

The oldest women continue to receive lower rates of optimal first-line therapy compared to younger women. Once adjusted for FIGO stage, surgical outcome and first-line treatment received, age was no longer an independent risk factor for poorer overall survival. Not receiving standard of care platinum-based chemotherapy and cytoreductive surgery would therefore appear to be a critical factor for the poorer survival outcomes seen in our oldest patients. In the absence of a formal geriatric or frailty assessment, using age alone may lead to inappropriate under-treatment, adversely affecting cancer outcomes in these women. Further assessment of the reasons behind the lower treatment rates in the oldest patients are essential to further understand and were beyond the scope of this retrospective study. Previous work by this group (manuscript under review) has demonstrated that older women desire active treatment and do not consider their age to be a hindrance. A formal frailty or geriatric assessment together with interventions to address issues identified would assist in optimising vulnerable patients. This could improve the rates of treatment delivery and completion in older adults thereby improving outcomes in this key demographic. The prospective UK FAIR-O study (NCT04300699) seeks to address the issue of assessment and management of frailty and medical comorbidities in the general oncology clinic.

## Figures and Tables

**Table 1:** Patient characteristics: stage and ECOG performance status at baseline.

	65–69 Years *n* = 91	70–74 Years *n* = 79	75–79 Years *n* = 53	>80 Years *n* = 57	Total *n* = 280	*p*
	*n* (%)	*n* (%)	*n* (%)	*n* (%)	*n* (%)	
**FIGO Stage**						
**1**	13 (14.3)	4 (5.1)	8 (15.1)	12 (21.1)	37 (13.2)	0.293
**2**	9 (9.9)	10 (12.7)	3 (5.7)	3 (5.3)	28 (10.0)	
**3**	52 (57.1)	44 (55.7)	33 (62.3)	29 (50.9)	158 (56.4)	
**4**	16 (17.6)	21 (26.6)	6 (11.3)	12 (21.1)	55 (19.6)	
**Unknown**	1 (1.1)	0	0	1 (1.8)	2 (0.7)	
**ECOG PS**						
**0**	30 (33.0)	12 (15.2)	9 (17.0)	5 (8.8)	56 (20.0)	0.008
**1**	34 (37.4)	38 (48.1)	27 (50.9)	23 (40.4)	122 (43.6)	
**2**	14 (15.4)	18 (22.8)	8 (15.1)	15 (26.3)	55 (19.6)	
**3**	5 (5.5)	9 (11.4)	3 (5.7)	9 (15.8)	26 (9.3)	
**Unknown**	8 (8.8)	2 (2.5)	6 (11.3)	5 (8.8)	21 (7.5)	
**Histological subtype**						
**High grade serous**	62 (68.1)	57 (72.2)	36 (67.9)	40 (70.2)	195 (69.6)	0.547
**Low grade serous**	3 (3.3)	3 (3.8)	3 (5.7)	3 (5.3)	12 (4.3)	
**Carcinosarcoma**	6 (6.6)	5 (6.3)	4 (7.5)	6 (10.5)	21 (7.5)	
**Clear cell**	8 (8.8)	1 (1.3)	2 (3.8)	1 (1.8)	12 (4.3)	
**Endometrioid**	7 (7.7)	3 (3.8)	2 (3.8)	2 (3.5)	14 (5.0)	
**Mucinous**	1 (1.1)	2 (2.5)	0	0	3 (1.1)	
**Adenocarcinoma/Mixed/ Undifferentiated**	4 (4.4)	8 (10.1)	6 (11.3)	5 (8.8)	23 (8.2)	

**Table 2:** Patient characteristics. Medical comorbidities and functional status at baseline.

	65–69 Years *n* = 91	70–74 Years *n* = 79	75–79 Years *n* = 53	>80 Years *n* = 57	Total *n* = 280	*p*
	***n* (%)**	***n* (%)**	***n* (%)**	***n* (%)**	***n* (%)**	
**Medical comorbidities**						
**Cardiovascular disease**	26 (28.6)	21 (26.6)	16 (30.2)	14 (24.6)	77 (27.5)	0.907
**Hypertension**	37 (40.7)	28 (35.4)	22 (41.5)	26 (45.6)	113 (40.4)	0.650
**Previous malignancy**	5 (5.5)	4 (5.1)	6 (11.3)	1 (1.8)	16 (5.7)	0.183
**Endocrine disease**	7 (7.7)	5 (6.3)	5 (9.4)	3 (5.3)	20 (7.1)	0.834
**Osteoarthritis**	4 (4.4)	5 (6.3)	7 (13.2)	4 (7.0)	20 (7.1)	0.252
**Rheumatological disease**	2 (2.2)	7 (8.9)	2 (3.8)	1 (1.8)	12 (4.3)	0.122
**CVA/MI/CAD**	7 (7.7)	4 (5.1)	6 (11.3)	3 (5.3)	20 (7.1)	0.512
**Haematological** **disease**	0	2 (2.5)	0	0	2 (0.7)	0.167
**Previous DVT**	15 (16.5)	8 (10.1)	8 (15.1)	4 (7.0)	35 (12.5)	0.345
**Polypharmacy (>3 meds)**	31 (34.1)	27 (34.2)	23 (43.4)	30 (52.6)	111 (39.6)	0.010
**Respiratory disease**	6 (6.6)	15 (19.0)	6 (11.3)	1 (1.8)	28 (10.0)	0.007
**Diabetes**	9 (9.9)	7 (8.9)	6 (11.3)	7 (12.3)	29 (10.4)	0.850
**Cognitive impairment**	0	2 (2.5)	0	6 (10.5)	8 (2.9)	0.001
**Depression**	6 (6.6)	4 (5.1)	0	1 (1.8)	11 (3.9)	0.193
**Functional baseline**						
**Lives alone**	20 (22.0)	31 (39.2)	18 (34.0)	29 (50.9)	98 (35.0)	0.000
**Lives in supported accommodation**	0	1 (1.3)	2 (3.8)	4 (7.0)	7 (2.5)	0.032
**Use of walking aids**	7 (7.7)	12 (15.2)	11 (20.8)	14 (24.6)	44 (15.7)	0.026
**Reduced activities of daily living**	16 (17.6)	22 (27.9)	13 (24.5)	19 (33.3)	70 (25.0)	0.226
**Assistance with activities of daily living**	7 (7.7)	10 (12.7)	8 (15.1)	10 (17.5)	35 (12.5)	0.441
**History of delirium in last 12 months**	0	0	0	3 (5.3)	3 (1.1)	0.007
**Cognitive impairment**	0	2 (2.5)	0	6 (10.5)	8 (2.9)	0.001
**Weight loss in last 3 months**	22 (24.2)	22 (27.9)	11 (20.8)	11 (19.3)	66 (23.6)	0.799
**Visual impairment**	3 (3.3)	1 (1.3)	2 (3.8)	9 (15.8)	15 (5.4)	0.016
**Hearing impairment**	1 (1.1)	0	2 (3.8)	3 (5.3)	6 (2.1)	0.242
**History of falls in last 12 months**	1 (1.1)	0	1 (1.9)	3 (5.3)	5 (1.8)	0.106

**Table 3:** Treatment tolerance.

	65–69 Years *n* = 82	70–74 Years *n* = 72	75–70 Years *n* = 48	>80 Years *n* = 39	Total *n* = 241	*p*-Value
	%	%	%	%	%	
Dose modification at baseline	9.8	12.5	6.3	17.5	11.6	0.365
Dose modification during chemotherapy	29.3	30.6	52.1	37.5	35.5	0.193
Completed 6 cycles of chemotherapy	86.6	86.1	77.1	65.0	82.2	0.034
≥ G2 Haematological toxicity	29.3	19.4	33.3	30.0	27.3	0.554
≥ G3 Non-haematological toxicity	13.4	19.4	27.1	32.5	21.1	0.082
Febrile neutropenia	4.9	2.8	2.1	0.00	2.9	0.540
Hospital admission during chemotherapy	20.7	34.7	25.0	37.5	28.5	0.135
Death within 30 days of chemotherapy	1.2	0.00	4.2	0.00	1.2	0.184

**Table 4:** Univariate and multivariate analysis of factors associated with poorer overall survival.

			Univariate			Multivariate		
		*n*	HR	95% CI	*p*	HR	95% CI	*p*
Age Cohort	65–69	91	-	-	-	-	-	-
	70–74	79	1.40	(0.96–2.05)	0.081	0.95	(0.60–1.52)	0.833
	75–79	53	1.07	(0.68–1.68)	0.772	0.79	(0.44–1.43)	0.441
	>80	57	2.20	(1.47–3.27)	0.000	1.76	(1.03–3.02)	0.04
FIGO Stage	1	37				-	-	-
	2	28	1.30	(0.55–3.07)	0.553	7.91	(0.98–63.61)	0.052
	3	158	3.69	(1.98–6.89)	0.000	12.99	(1.77–95.20)	0.012
	4	55	6.00	(3.08–11.68)	0.000	16.16	(2.11–123.61)	0.007
ECOG PS	0	56				-	-	-
	1	122	1.86	(1.18–2.93)	0.007	2.14	(1.21–3.79)	0.009
	2	55	4.02	(2.47–6.53)	0.000	2.53	(1.20–5.35)	0.015
	3	26	7.36	(4.13–13.13)	0.000	3.51	(1.37–8.99)	0.009
Cardiovascular disease		77	1.38	(1.01–1.90)	0.043	0.99	(0.62–1.57)	0.95
Taking 3 or more medications		111	0.07	(0.01–0.55)	0.011	1.12	(0.72–1.74)	0.62
Osteoarthritis		16	1.70	(0.96–3.01)	0.070	1.62	(0.76–3.44)	0.209
Reduced activities of daily living		70	2.89	(2.10–3.98)	0.000	1.53	(0.90–2.62)	0.118
History of depression		11	1.86	(0.95–3.65)	0.071	1.89	(0.83–4.30)	0.128
History of weight loss		66	1.51	(1.09–2.11)	0.015	0.93	(0.59–1.47)	0.754
Albumin <35 g/L		114	2.09	(1.56–2.81)	0.000	1.52	(0.97–2.38)	0.065
Haemoglobin <120 g/L		141	1.28	(0.9601.72)	0.093	0.80	(0.53–1.23)	0.311
GFR <60 mL/min		104	1.33	(0.98–1.79)	0.064	1.11	(0.74–1.68)	0.607

**Table 5:** Univariate and multivariate factors associated with poorer overall survival.

		Univariate			Multivariate		
		HR	95% CI	*p*	HR	95% CI	*p*
Age Cohort	65–69 years	-	-	-	-	-	-
	70–74 years	1.40	(0.96–2.05)	0.081	1.22	(0.77–1.95)	0.386
	75–79 years	1.07	(0.68–1.68)	0.772	0.85	(0.46–1.54)	0.582
	>80 years	2.20	(1.47–3.27)	0.000	0.81	(0.40–1.61)	0.541
FIGO stage	1				-	-	-
	2	1.30	(0.55–3.07)	0.553	2.39	(0.86–6.64)	0.094
	3	3.69	(1.98–6.89)	0.000	4.38	(2.02–9.52)	0.000
	4	6.00	(3.08–11.68)	0.000	6.96	(2.81–17.24)	0.000
Surgical outcome	Residual disease	2.71	(1.82–4.03)	0.000	2.09	(1.32–3.30)	0.002
	Platinum- combination	-	-	-	-	-	-
Chemotherapy	Single-agent carboplatin	1.29	(0.93–1.77)	0.123	1.34	(0.85–2.17)	0.203
	No chemotherapy	2.19	(1.43–3.35)	0.000	4.49	(1.99–10.13)	0.000
	Completed 6 cycles	0.34	(0.23–0.49)	0.000	0.33	(0.19–0.59)	0.000

## Data Availability

The data presented here are available on request from the corresponding author.
